# Modern Technique of the Cæsarian Section

**Published:** 1897-12

**Authors:** Barton Cook Hirst

**Affiliations:** Philadelphia, Pa.; Professor of Obstetrics in the University of Pennsylvania


### MODERN TECHNIQUE OF THE CÆSARIAN SECTION.

BY BARTON COOK HIRST, A. M., M. D., PHILADELPHIA, PA.

Professor of Obstetrics in the University of Pennsylvania.

Cæsarian section being an operation destined to become a quite frequent procedure, it may not be inopportune to outline the method best calculated to give desirable results.

PREPARATION.—On admission to the hospital (if the work is to be done under the more favorable surroundings of a modern hospital) the patient should be given a warm bath. She should then be put in bed and kept there until the operation is to be performed. The following prescription should be given.

| | |
|---|---|
| ℞. Strychninæ, sulph | 0.06 |
| Pulv. digitalis | 0.6 |
| Quininæ sulph. | 1.5 |

Misce et ft. capsul XII. Sig.: One t. i. d.

A good movement of the bowels must be secured daily—as by two drams of Rochelle salts each evening.

The heart, lungs and urine should each be carefully examined.

On the day before operation a diet as follows is to be ordered: Gruel for breakfast, soup for dinner, milk toast for supper, one glass of milk 10 a. m., to 4 p. m., and 9 p. m.

At 5 p. m., on the afternoon before the operation, ten grains sulphonal in half glass of boiling water cooled down to temperature that permits of its being drunk, may be given if patient is nervous and has been sleepless. At 9 p. m., half ounce Epsom salts in a tumblerful of water.

The evening before the operation the nurse is to be given the following instructions for the preliminary cleaning:

1. Sterilize the following articles for twenty minutes at 240 degrees: Soft bristle brush, absorbent cotton, one-half dozen towels, gauze unmedicated, binder, long gown.

2. The resident physician, or under his supervision the nurse who cleanses the abdomen, must prepare his or her hands and arms as though about to operate, namely, remove rings, scrub with brush, hot water and tincture of green soap for ten minutes; clean nails with nail file. Scrub hands and arms with benzine and then with alcohol, immerse hands and arms in bichloride solution (1:1000) for two minutes. Then put on the sterilized gown.

3. The abdomen from ensiform to symphysis and from flank to flank must be scrubbed with soft bristle brush, tincture of green soap and hot water thoroughly (for at least ten minutes), paying special attention to navel and to pubic regions. Wipe off the razor with cotton and alcohol. Shave pubis, and then scrub thoroughly with alcohol. Cover up the abdomen with the sterile gauze and put on the binder.

4. On the morning of the operation give cup beef tea at 7 a. m. Hands of nurse or doctor are to be cleansed as described above. Articles resterilized as described above, same cleansing of abdomen repeated as described, but in addition before alcohol scrubbing, scrub the abdomen with benzine. Wring out a sterile towel in 1:1000 bichloride solution, and cover the abdomen with the towel, put over it a thick layer of sterile cotton; and apply binder. Catheterize the woman just before anesthetization with a sterile glass catheter in an aseptic manner. Give a vaginal douche, one quart of 1:4000 sublimate solution followed by a little sterile water. If the bowels have not opened freely, give an enema, a pint of soapsuds with one dram of turpentine.

THE OPERATION.—With a large scalpel held firmly in the full hand a free incision is made from two inches above the umbilicus to just above the symphysis. This incision may be carried entirely through the abdominal wall in its upper part, as the intestines are out of the way. The abdominal opening is then enlarged with scissors downward as low as possible. An assistant makes the wound gape while the operator delivers the womb from the abdominal cavity. The assistant then approximates the edges of the abdominal wound as closely as possible around and above the cervix, at the same time squeezing the latter with the outspread hands. With a few rapid, but light strokes of the knife the operator makes an incision an inch in length, through the uterine muscle, and not through the membrane, so as not to cut the child. Then with one rapid movement of the left hand and arm, the uterine wall is torn down to the internal os, the membranes are ruptured, the placenta if in the way is detached and pushed aside, the child is seized by the most accessible part (either shoulder or leg), is delivered and with the placenta still attached to it is dropped into a sterile sheet spread over the outstretched arms of an assistant, who stands directly at the surgeon's left hand, and whose duty it is to revive the child if it is asphyxiated, and to tie and cut the cord.

Up to this point the operation rarely requires seventy-five seconds. Then follows an easy hysterectomy, ligation of the ovarian clamps, cutting the broad ligaments, preparation of the peritoneal flaps, and amputation of the womb, ligation of the uterine arteries, and over-sewing of the stump which it dropped.

The abdominal wall may be closed by close-set interrupted stitches—the easiest plan for a beginner, or by any other method that a more experienced operator may prefer.—*Am. Jour. Surg. & Gyn.*

